# Functional analysis of Samd11, a retinal photoreceptor PRC1 component, in establishing rod photoreceptor identity

**DOI:** 10.1038/s41598-021-83781-1

**Published:** 2021-02-18

**Authors:** Shun Kubo, Haruka Yamamoto, Naoko Kajimura, Yoshihiro Omori, Yamato Maeda, Taro Chaya, Takahisa Furukawa

**Affiliations:** 1 Laboratory for Molecular and Developmental Biology, Institute for Protein Research, Osaka University, 3-2 Yamadaoka, Suita, Osaka 565-0871 Japan; 2 Research Center for Ultrahigh Voltage Electron Microscopy, Osaka University, Osaka, 567-0047 Japan

**Keywords:** Neuroscience, Development of the nervous system, Visual system

## Abstract

Establishing correct neuronal cell identity is essential to build intricate neural tissue architecture and acquire precise neural function during vertebrate development. While it is known that transcription factors play important roles in retinal cell differentiation, the contribution of epigenetic factors to establishing cell identity during retinal development remains unclear. We previously reported that Samd7, a rod photoreceptor cell-specific sterile alpha motif (SAM) domain protein, functions as a Polycomb repressive complex 1 component (PRC1) that is essential for establishing rod identity. In the current study, we analyzed a functional role of Samd11*,* another photoreceptor-enriched SAM-domain protein*,* in photoreceptor differentiation and maturation. We observed that Samd11 interacts with Phc2 and Samd7, suggesting that Samd11 is a component of PRC1 in photoreceptor cells. We generated *Samd11*-null allele and established *Samd7/11* double knock-out (DKO) mouse. The *Samd7/11* DKO retina exhibits shortened photoreceptor outer segments by electron microscopy analysis. Microarray analysis revealed that *Samd7/11* DKO up-regulated more retinal genes than *Samd7*^*−/−*^ alone, partial functional redundancy of *Samd7* and *Samd11*. Taken together, the current results suggest that Samd7 and Samd11 are PRC1 components and that Samd7 is the major regulator while Samd11 is an accessory factor used for the establishment of precise rod photoreceptor identity.

## Introduction

Acquiring precise cell identities for a large diversity of neurons and glial cells is essential to form the complicated central nervous system (CNS) structure and neural function during vertebrate development. The vertebrate retina is a part of the CNS and comprised of five major types of neurons (photoreceptor, bipolar, horizontal, amacrine, and ganglion cells) and Müller glial cells. Retinal photoreceptor cells are composed of rods and multiple types of cones. While rods express rhodopsin and mediate scotopic vision, cones express cone opsin and are responsible for photopic and color vision. Each cone subtype expresses a spectrally-sensitive cone opsin that gathers information about light wavelength. Rod and cone subtype-specific genes, including opsins and phototransduction component genes, are modified during retinal photoreceptor development for subtype-specific expression. This allows proper retinal function and normal rod and cone cell morphology. Aberrant gene expression in photoreceptor cells is thought to contribute to the development of retinal degenerative diseases, including retinal pigmentosa and cone dystrophy^1^. Regulating subtype-specific gene expression during photoreceptor development appears to be associated with epigenetic mechanisms. A previous study reported that in rods the *S-opsin* genomic locus exhibits low H3K27me3 levels at postnatal day 2 (P2), a period when rod photoreceptors are still immature and express *S-opsin*^2^. However, during adulthood, the *S-opsin* locus showed increased H3K27me3 levels and suppressed S-opsin expression in mature rods. Different epigenetic landscapes between rods and cones have also been reported^3,4^. Compared to the cone nuclei, the rod nuclei exhibit inverted architecture, with the heterochromatin localized in the center and the euchromatin localized in the periphery of the mouse retina^5,6^. In mature cones, the fetal enhancers that are active in retinal progenitor cells become methylated to suppress expression of the progenitor genes, while the fetal enhancers in mature rods remain unmethylated^3^. We previously showed that a rod-specific Polycomb repressive complex (PRC) 1 component, Samd7, is essential for H3K27me3 and H2AK119Ub regulation to establish rod identity^7^.

We previously identified *Samd11(mr-s)* while screening for molecules involved in photoreceptor development and/or maintenance^8^. The SAM domains of Samd7 and Samd11 show an amino acid identity of 69%. Samd7 and Samd11 exhibit dominant expression in developing and mature photoreceptor cells. In the present study, we investigated the function of *Samd11* during the establishment of rod photoreceptor identity.

## Results

### *Samd11* alone is not essential for retinal development

**Figure 1:**
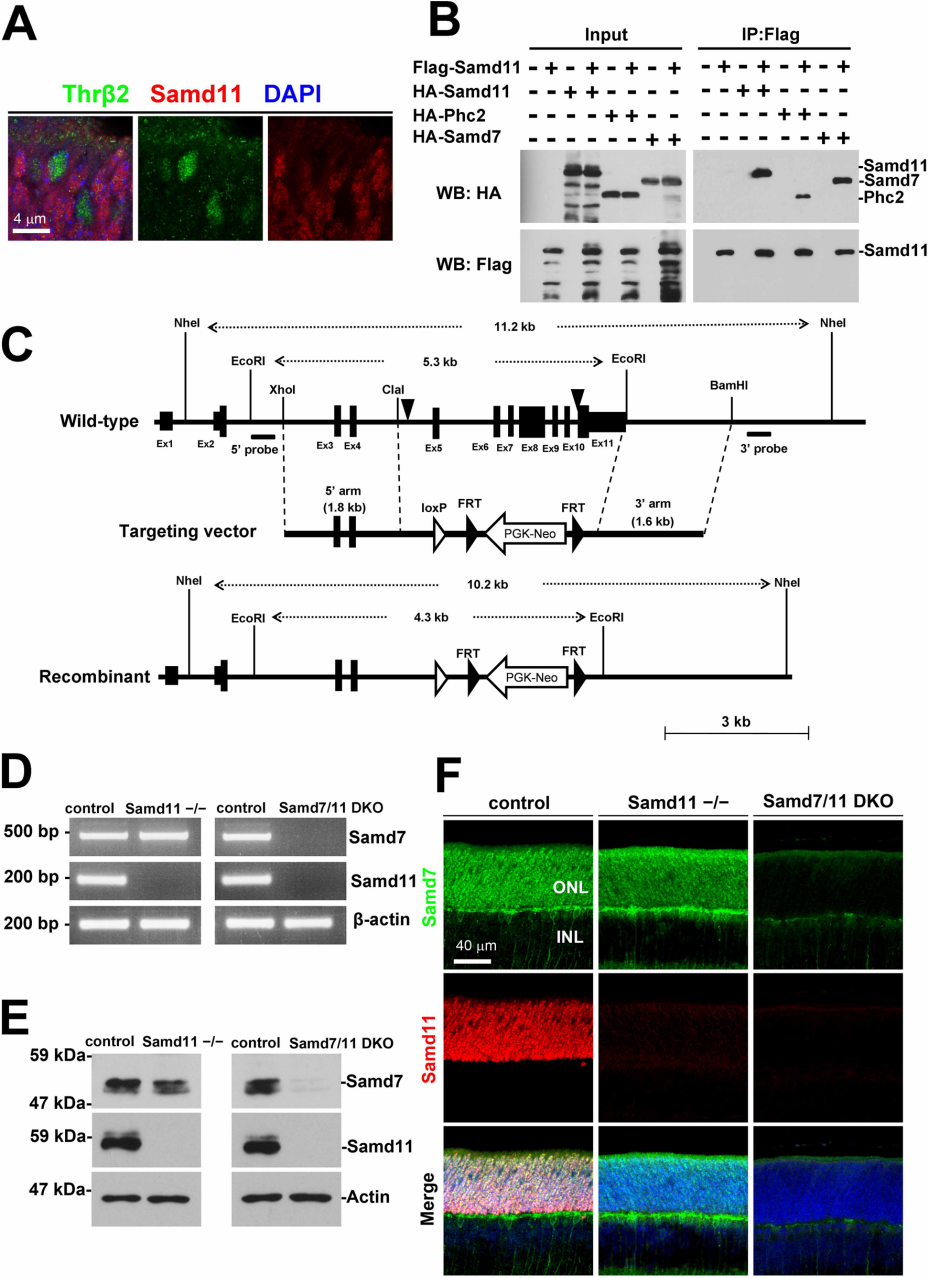
Samd11 expression in the mouse retina and generation of the *Samd11*^*−/−*^ allele. **(A**) Retinal sections from P4 WT mice were immunostained using the antibodies against Samd11 (red) and Thrβ2 (a cone photoreceptor cell marker, green) with DAPI (blue). Samd11 signals did not overlap with Thrb2-positive cells. **(B)** Immunoprecipitation analysis of Samd11/Samd11, Samd11/Phc2, and Samd11/Samd7. HEK293 cells were transfected with FLAG-tagged Samd11-expressing plasmid paired with HA-tagged Samd11-, Phc2- or Samd7-expressing plasmid. Samd11 interacted with Samd11, Phc2, and Samd7. Full-length blots are presented in Figure S1. **(C)** A schematic diagram of the targeted deletion of the *Samd11* gene. Exons 5 to 11 were replaced with the targeting vector. The arrowheads indicate CRISPR/Cas9 cut points. The 5ʹ and 3ʹ probes used to confirm homologous recombination by southern blot are indicated. **(D)** RT-PCR analysis of *Samd7* and *Samd11* transcription using intron-spanning primer sets in *Samd11*^*−/−*^ and *Samd7/11* DKO retinas at P12. The 467 bp *Samd7* fragment and 187 bp *Samd11* fragment were amplified from the control retina. No significant *Samd7* transcript was detected in the *Samd7/11* DKO retina. No significant *Samd11* transcript was detected in the *Samd11*^*−/−*^ or *Samd7/11* DKO retina. β*-actin* was used as a loading control. The 200 bp and 500 bp positions for DNA size marking are indicated. Full-images are presented in Figure S1. (E) Western blot analysis of Samd7 and Samd11 proteins in *Samd11*^*−/−*^ and *Samd7/11* DKO retinas at P12. The approximate molecular weights for the Samd7 band (53 kDa) and Samd11 band (57 kDa) were detected in the control retina, but not in their respective knockouts. β-actin was used as a loading control. The molecular protein weights for 59 kDa and 47 kDa are indicated. Full-length blots are presented in Figure S1. **(F)** Immunostaining of control, *Samd11*^*−/−*^, and *Samd7/11* DKO mice retinal sections at P9 using anti-Samd7 (green) and anti-Samd11 antibodies (red) with DAPI (blue). No Samd11 signal was detected in the photoreceptor layer of the *Samd11*^*−/−*^ retina. The Samd7 and Samd11 signals were undetected in the *Samd7/11 DKO* retina.

Our previous study showed that *Samd11* expression peaks at the stage of rod photoreceptor differentiation is proceeding^8^, similar to the *Samd7* expression pattern. A yeast two-hybrid screen using Samd7 as bait indicated that Samd7 interacts with Samd11^7^. These observations suggest that Samd11 and Samd7 have functional similarity. We investigated the functional role and mechanism of *Samd11* in retinal development. First, to examine the cellular localization of Samd11, we immunostained P4 mouse retinal sections with the anti-Samd11 antibody that we generated and the anti-Thyroid hormone receptor β2 (Thrβ2) antibody^9^ as a marker for cone photoreceptor nuclei. While the Samd11 signal was enriched in rods in the ONL, no significant signal was detected in cones (Fig. 1A). Our previous study showed that Samd7 forms a complex with PRC1 by interacting with Phc2 to repress target gene expression through H3K27 me3 and H2AK119ub marks^7^, which PRCs are known to regulate^10^. We performed an immunoprecipitation assay to investigate the interaction between Samd11 and Phc2. We confirmed homophilic Samd11/Samd11 interactions and heterophilic Samd11/Samd7 and Samd11/Phc2 interactions (Fig. 1B and Fig. S1A), suggesting that Samd11 is a PRC1 component in rods.

In order to investigate the *Samd11* function in vivo*,* we generated *Samd11*^*−/−*^ mice by targeted gene disruption (Fig. 1C) to produce a total SAM domain deletion. The loss of *Samd11* mRNA and Samd11 protein in *Samd11*^*−/−*^ retinal sections was confirmed by RT-PCR, western blot, and immunostaining (Fig. 1D–F and Fig. S1B, S1C). *Samd11*^*−/−*^ mice were viable and showed no gross abnormalities. We also generated *Samd7/11* DKO mice to investigate the suspecting functional redundancies of *Samd7* and *Samd11* during retinal development. *Samd7/11* DKO mice were born in Mendelian ratios, viable, and fertile, with no apparent morphological abnormalities. Immunostaining the retinal sections of *Samd7/11* DKO mice using the anti-Samd7 and Samd11 antibodies revealed that Samd7 and Samd11 signals were absent in the *Samd7/11* DKO retina. We also confirmed the loss of *Samd7*/*11* mRNA and protein in the retina using RT-PCR and western blot, respectively (Fig. 1D–F and Fig. S1B, S1C).

### *Samd7/11* DKO mice show photoreceptor outer segment disorganization

**Figure 2:**
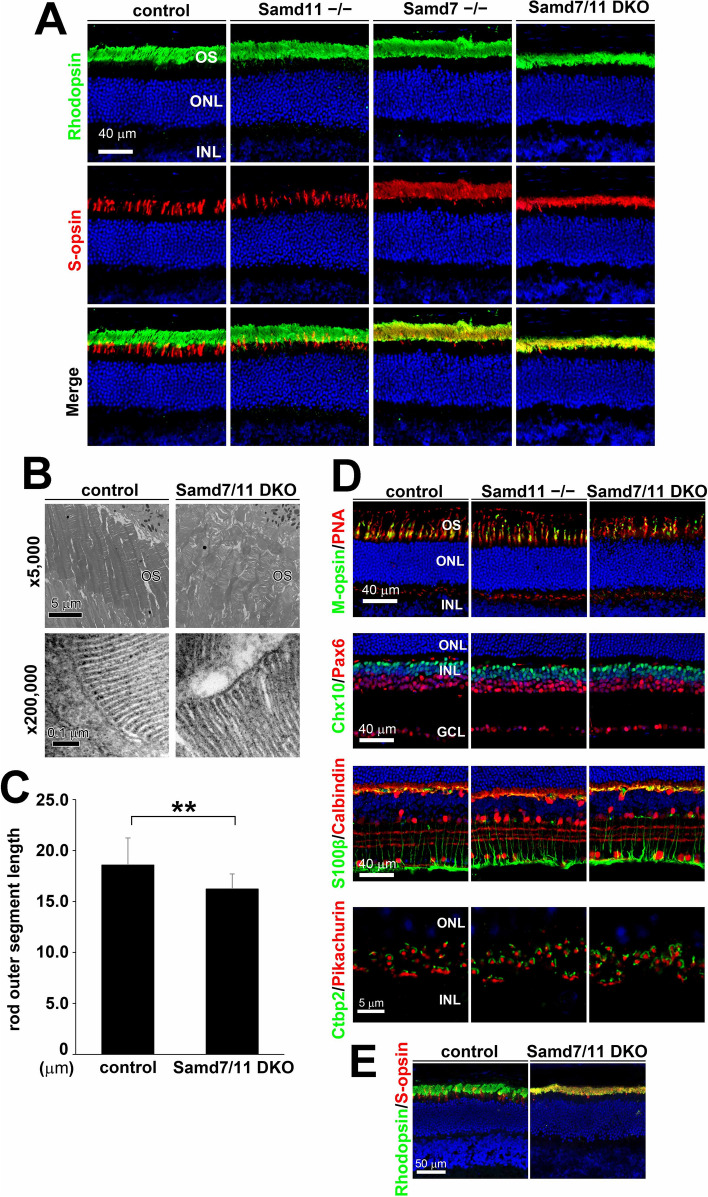
Immunohistochemical analysis of *Samd11*^*−/−*^ and *Samd7/11* DKO retinas. **(A)** Immunostaining of 2 M control, *Samd11*^*−/−*^, *Samd7*^*−/−*^, and *Samd7/11* DKO retinal sections using anti-Rhodopsin (green) and anti-S-opsin antibodies (red) with DAPI (blue). **(B,C)** TEM analysis of outer segments at 2 M. **(B)** The outer segments observed in *Samd7/11* DKO retinas were disorganized, but the photoreceptor disk structure of the outer segments was unaffected compared to the control retina. **(C)** Measuring the rod photoreceptor cells’ outer segments length showed significantly shorter outer segments in the *Samd7/11* DKO retina compared to that in the control retina. Error bars indicate mean ± SD, n = 30 from three retinas per group. Student’s t-test; **P < 0.01.** (D)** Immunostaining of 2 M control, *Samd11*^*−/−*^, *Samd7*^*−/−*^, and *Samd7/11* DKO retinal sections using multiple antibodies. Retinas were stained with anti-M-opsin, a cone outer segment marker (green); PNA, a marker of cone outer and inner segments and cone synaptic terminals (red); anti-Pax6, an amacrine and ganglion cells marker (red); anti-Chx10, a bipolar cell marker (green); anti-S100β, a Müller glia marker (green); anti-Calbindin, an amacrine and horizontal cell marker (red); anti-Ctbp2, a synaptic ribbons marker (green); anti-Pikachurin, a photoreceptor synaptic cleft marker (red); and DAPI (blue). **(E)** Immunostaining of 12 M control and *Samd7/11* DKO retinal sections using anti-Rhodopsin (green) and anti-S-opsin antibodies (red) with DAPI (blue). No substantial change was observed in the ONL thickness.

The *Samd7*^*−/−*^ retina displays strong ectopic S-opsin expression in rods^7^. Therefore, we first examined photoreceptor subtype-specific opsin expression in wild-type (WT) control, *Samd11*^*−/−*^, and *Samd7/11* DKO retinas. We immunostained blue-cone and rod photoreceptor outer segments in the Two-month-old (2 M) mouse retina using anti-S-opsin and anti-Rhodopsin antibodies. S-opsin signals were detected in a discrete pattern in the control retina. However, the *Samd7/11* DKO retina showed strong S-opsin signals in rod and cone outer segments, similar to the pattern reported in the *Samd7*^*−/−*^ retina (Fig. 2A). In contrast, S-opsin and Rhodopsin signals were not substantially different between the *Samd11*^*−/−*^ and control retinas. The ONL thickness did not differ between the *Samd7/11* DKO and control retinas, indicating that photoreceptor degeneration was not observed until 2 M. In contrast, the *Samd7/11* DKO retina had a reduced outer segment thickness in addition to the strong S-opsin signals (Fig. 2A).

Further investigation of outer segment structural integrity with TEM analysis revealed disorganized outer segments in the *Samd7/11* DKO retina (Fig. 2B). We observed wider and more loosely packed discs in the *Samd7/11* DKO retina than those in the WT control retina. The *Samd11*^*−/−*^ and *Samd7*^*−/−*^ retinas exhibited similar outer segment morphologies compared to those in the control retina (Fig. S2). We next measured the length of the rod outer segments. Although the photoreceptor disc structure morphology was unaffected in the *Samd7/11* DKO retina, the rod outer segment length was significantly decreased by approximately 10% compared to that in the control retina (Fig. 2B, C; Student’s *t*-test: *P <* 0.001).

Next, we immunostained the control, *Samd11*^*−/−*^, and *Samd7/11* DKO retinas with PNA, a marker of cone outer and inner segments and synaptic terminals, and anti-M-opsin, a green-cone outer segment marker (Fig. 2D). PNA and M-opsin signals in the *Samd11*^*−/−*^ and *Samd7/11* DKO retinas were not substantially different from the control retina. To investigate the integrity of non-photoreceptor cells in the *Samd11*^*−/−*^ and *Samd7/11* DKO retinas, we immunostained photoreceptor synaptic ribbons using an anti-Ctbp2 and anti-Pikachurin antibodies, photoreceptor synaptic terminal markers. These markers were not significantly changed in *Samd11*^*−/−*^ and *Samd7/11* DKO retinas compared to controls (Fig. 2D). We also examined the retinas using anti-Chx10 (a bipolar cell marker), anti-Pax6 (an amacrine and ganglion cell marker), anti-S100β (a Müller glia cell marker), and anti-Calbindin (a horizontal and amacrine cell marker) antibodies. The *Samd11*^*−/−*^ and *Samd7/11* DKO retinas showed no significant differences in these immunostained signals compared to the control retina at 2 M (Fig. 2D). We immunostained the control, *Samd11*^*−/−*^, *Samd7*^*−/−*^, and *Samd7/11* DKO retinas using anti-H3K4me3 (a marker for euchromatin) and anti-Lamin B (a marker for nuclear membrane) antibodies to visualize the rod nucleus. We examined the immunostained images, and no obvious changes of rod euchromatin morphology in the control, *Samd11*^*−/−*^, *Samd7*^*−/−*^, or *Samd7/11* DKO retinas were noted (Fig. S3). Since the *Samd7/11* DKO mice showed shortened outer segments, we also examined ONL thickness using DAPI staining to investigate whether photoreceptor degeneration occurred in the *Samd7/11* DKO retina. We did not observe significant degeneration in the *Samd7/11* DKO retina at 12 M (Fig. 2E).

### Loss of *Samd7* and *Samd11* affects rod response to light

**Figure 3:**
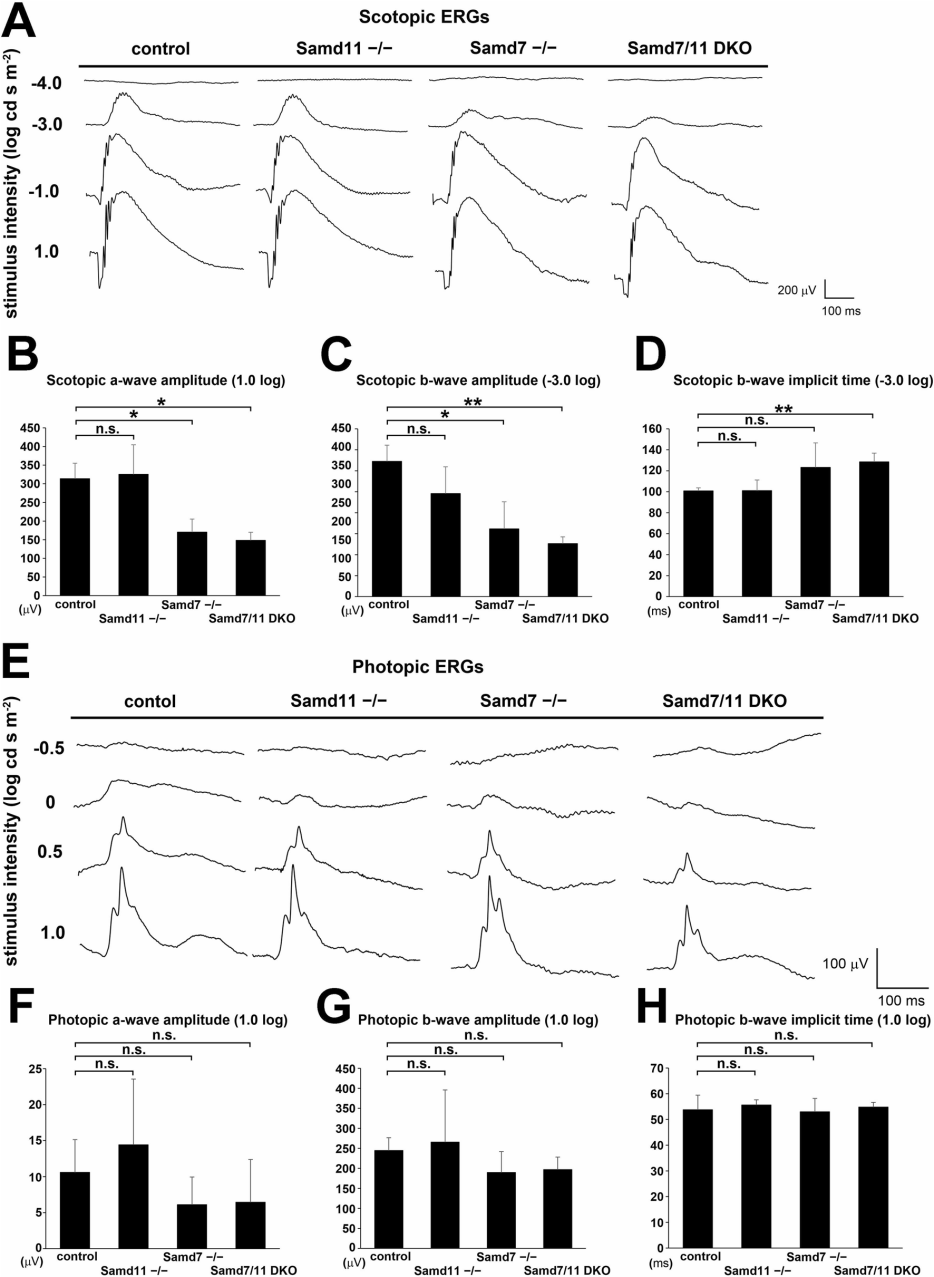
ERG analysis of *Samd11*^*−/−*^ and *Samd7/11* DKO mice. **(A)** Representative scotopic ERGs recorded from 2 M control, *Samd11*^*−/−*^, *Samd7*^*−/−*^, and *Samd7/11* DKO mice under a range of white light. **(B)** The mean scotopic ERG a-wave amplitudes. The *Samd7*^*−/−*^, and *Samd7/11* DKO mice showed reduced amplitudes at 1.0 log cd sm^-2^. **(C)** The mean scotopic ERG b-wave amplitudes. The *Samd7*^*−/−*^, and *Samd7/11* DKO mice showed reduced amplitudes at − 3.0 log cd sm^−2^. **(D)** The implicit time of the scotopic ERG b-wave was prolonged in the *Samd7/11* DKO mice at − 3.0 log cd sm^−2^. (**E**) Representative photopic ERGs recorded from 2 M control, *Samd11*^*−/−*^, *Samd7*^*−/−*^, and *Samd7/11* DKO mice under a range of white light. **(F)** The mean photopic ERG a-wave amplitudes elicited by 1.0 log cd-s/m^2^ white-light stimuli. **(G)** The mean photopic ERG b-wave amplitudes elicited by 1.0 log cd-s/m^2^ white-light stimuli. **(H)** The implicit time of the photopic ERG b-wave elicited by 1.0 log cd-s/m^2^ white-light stimuli. Error bars indicate mean ± SD, n = 3 per group. Student’s t-test; **P < 0.01, *P < 0.05, *n.s.* not significant.

*Samd7*^*−/−*^ mice showed lower sensitivity against low-to-moderate flash luminescence than control mice^7^. To investigate the physiological role of *Samd11* in the retina, we measured ERGs in control, *Samd7*^*−/−*^, *Samd11*^*−/−*^, and *Samd7/11* DKO mice at 2 M (Fig. 3A–E). The dark-adapted (scotopic) ERGs elicited by four different white light stimuli intensities (− 4.0, − 3.0, − 1.0, + 1.0 log cd sm^−2^) were measured (Fig. 3A). The a-wave amplitude, which indicates rod activity, was significantly decreased in the *Samd7*^*−/−*^ and *Samd7/11* DKO mice, but not *Samd11*^*−/−*^ mice, compared to the control mice at + 1.0 log cd sm^−2^ (Fig. 3B; Student’s *t*-test: *Samd7*^*−/−*^, *P* = 0.010; *Samd7/11* DKO, *P* = 0.004). The b-wave amplitude, which indicates the activity of rod bipolar cells activated by rods, was significantly decreased in *Samd7*^*−/−*^ and *Samd7/11* DKO mice compared to controls at − 3.0 log cd sm^−2^ (Fig. 3C; Student’s *t*-test: *Samd7*^*−/−*^*, P* = 0.021;* Samd7/11* DKO, *P* = 0.001). The b-wave amplitude tended to decrease in *Samd7/11* DKO mice compared to *Samd7*^*−/−*^ mice at − 3.0 log cd sm^−2^. Moreover, the implicit time of the b-wave, which indicates the transduction process speed between rods and bipolar cells, was significantly delayed in *Samd7/11* DKO mice compared to controls at − 3.0 log cd sm^−2^ (Fig. 3D; Student’s *t*-test: *P* = 0.005).

Next, we analyzed light-adapted (photopic) ERGs, which primarily represent cone activity, elicited by white light stimuli at four different intensities (− 0.5, 0, + 0.5, + 1.0 log cd sm^−2^) (Fig. 3E). The amplitudes and implicit times of photopic a-waves and b-waves did not significantly differ between control, *Samd11*^*−/−*^, and *Samd7/11* DKO mice (Fig. 3F–H). These results suggest that loss of *Samd11* alone does not affect the retinal physiological function. However, *Samd7/11* DKO mice showed lower sensitivity to low-to-moderate flash luminescence than control mice. In addition, *Samd7/11* DKO mice displayed delayed signal transmission from rods to rod bipolar cells at low flash luminescence compared to control mice, which was not observed in *Samd7*^*−/−*^ mice.

### *Samd11*^*−/−*^ and *Samd7/11* DKO retinal gene expression profiles

**Figure 4:**
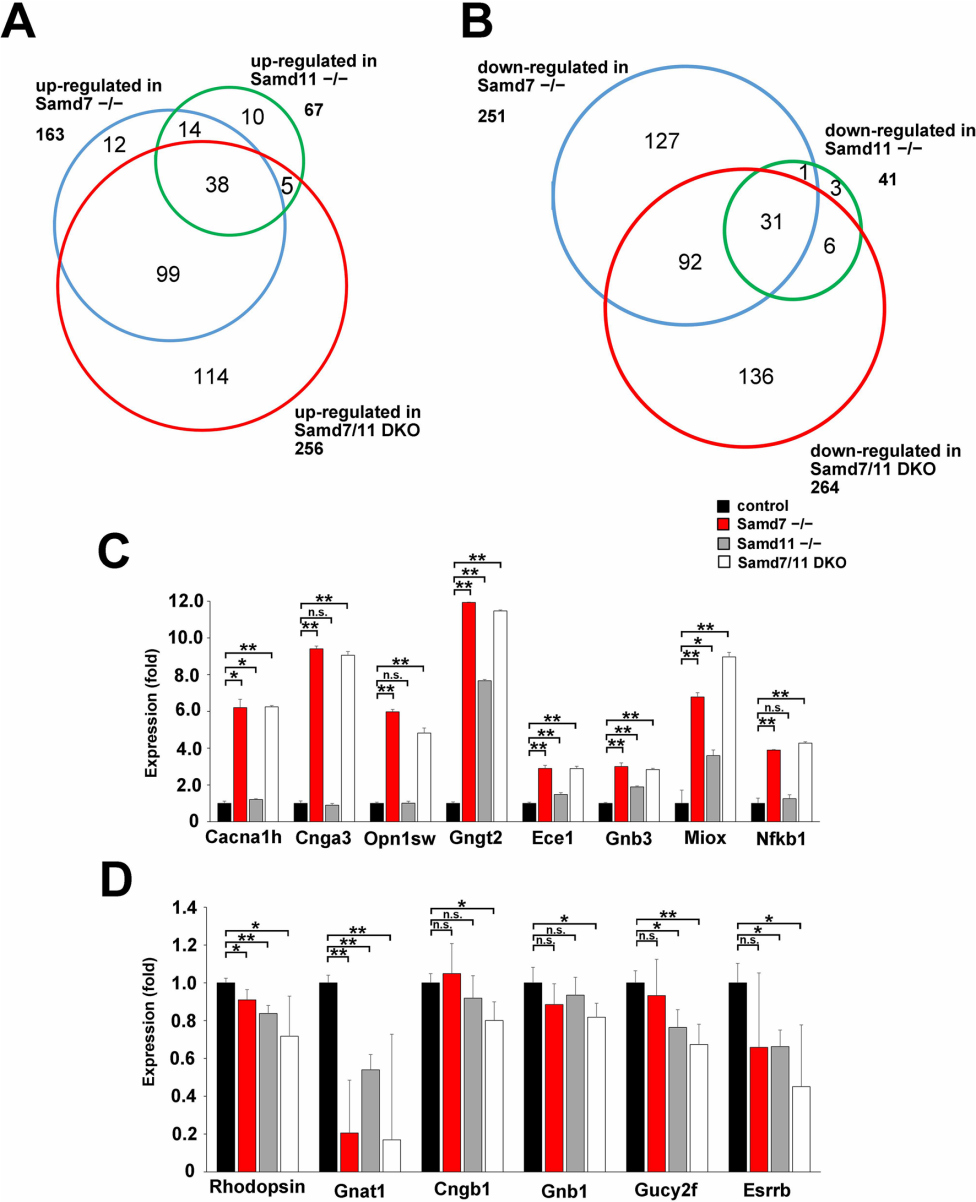
Retinal gene expression profiles in *Samd7*^*−/−*^, *Samd11*^*−/−*^, and *Samd7/11* DKO mice. **(A,B)** Venn diagram of up-regulated **(A)** and down-regulated **(B)** genes in *Samd7*^*−/−*^, *Samd11*^*−/−*^, and *Samd7/11* DKO retinas compared to the genes expressed in the control retina. Microarray analysis was performed using mRNA from control, *Samd11*^*−/−*^, and *Samd7/11* DKO retinas at P12. The number of up-regulated genes (signal log ratio greater than + 1.0) in the *Samd11*^*−/−*^, retina (green circle) and *Samd7/11* DKO retina (red circle) was 67 and 256, respectively, compared to the control retina. The number of down-regulated genes (signal log ratio less than − 0.5) in the *Samd11*^*−/−*^ retina (green circle) and *Samd7/11* DKO retina (red circle) were 41 and 264, respectively, compared to the control retina. **(C,D)** The up-regulation **(C)** and down-regulation **(D)** of selected genes from the microarray analysis was confirmed by qRT-PCR with mRNA from control, *Samd11*^*−/−*^, *Samd7*^*−/−*^, and *Samd7/11* DKO retinas at P12. Error bars indicate mean ± SD, n = 3 per group. Student’s t-test; **P < 0.01, *P < 0.05, *n.s* not significant.

To investigate retinal genome-wide gene expression profiles, we performed DNA microarray analysis on control, *Samd11*^*−/−*^, and *Samd7/11* DKO retinas at P12. We compared these gene expression profiles with the *Samd7*^*−/−*^ retinal profile obtained in our previous study^7^. Compared to the control retina, we identified 67 up-regulated (signal log ratio greater than + 1.0) and 41 down-regulated genes (signal log ratio less than − 0.5) in the *Samd11*^*−/−*^ retina, whereas we identified 256 up-regulated and 264 down-regulated genes in the *Samd7/11* DKO retina (Fig. 4A,B). The number of up- and down-regulated genes was increased in the *Samd7/11* DKO retina compared to that in the *Samd7*^*−/−*^ retina (Fig. 4A,B).

Consistent with the microarray analysis results, we confirmed that the expression of several genes was markedly altered in *Samd11*^*−/−*^ and *Samd7/11* DKO retinas compared to that in the control retinas (Fig. 4C,D). *Myo-inositol oxygenase (Miox)* was significantly up-regulated in the *Samd7/11* DKO retina compared to that in the *Samd7*^*−/−*^ retina (Fig. 4C; Student’s *t*-test: *Samd7*^*−/−*^, *P* = 0.004; *Samd7/11* DKO, *P* = 0.027; *Samd7/11* DKO, *P* = 0.004). qRT-PCR confirmed that the rod-related genes, *Cngb1* and *Gnb1,* were significantly down-regulated in the *Samd7/11* DKO, but not *Samd7*^*−/−*^, retina compared to the control retina (Fig. 4D; Student’s *t*-test: *Samd7/11* DKO, *P* = 0.020; *Samd7/11* DKO, *P* = 0.036, respectively).

## Discussion

The current study generated and analyzed *Samd11*^*−/−*^ and *Samd7/11* DKO mice. While *Samd11*^*−/−*^ mice displayed no overt retinal phenotype, *Samd7/11* DKO mice showed a severer defect in photoreceptor morphogenesis and altered gene expression profiles compared to *Samd7*^*−/−*^ mice. In terms of gene expression, *Samd7/11* DKO mice exhibited substantially increased number of up-regulated genes in the retina compared to *Samd7*^*−/−*^ mice. The amino acid identity of the SAM domain between Samd7 and Samd11 is relatively high, and Samd7 and Samd11 expressions are restricted to developing and mature photoreceptors. These observations suggest that Samd7 is the major regulator while Samd11 is an accessory factor used for the establishment of precise rod photoreceptor identity. The microarray analysis revealed that *Samd7* expression is about 2.7 times higher than *Samd11* expression. This may suggest that *Samd7* and *Samd11* expression levels reflect their functional activity, resulting in no obvious retinal histology and ERG phenotypes in the *Samd11*^*−/−*^ mice.

We observed disorganized outer segments in the *Samd7/11* DKO retina using TEM analysis. The microarray analysis of control and *Samd7/11* DKO retinas revealed multiple up-regulated genes involved in cone phototransduction and down-regulated genes related to rod phototransduction, including *Cnga3, Opn1sw, Cngb1,* and *Rhodopsin.* We re-examined the 136 down-regulated genes and 114 up-regulated genes of the *Samd7/11* DKO retina in our microarray data; however, we did not identify any gene that could be involved in rod outer segment formation, including *Rom1* and *Peripherin*. These observations suggest that other genes, whose functions remain unknown, might be involved in the shortened outer segments in the *Samd7/11* DKO retina. There is also the possibility that outer segment disorganization is caused by a combinatory effect of multiple affected genes or secondary effects of the gene expression changes in the *Samd7/11* DKO retina. There is another possibility that outer segment disorganization might be caused by a combined effect of multiple affected genes or secondary effects of gene expression changes in the *Samd7/11* DKO retina.

What is the biological significance of the Samd7/11 function in association with PRC1 for establishing rod photoreceptor identity? There are hundreds of different SAM-domain proteins across various species from humans to yeasts. Yet, to our knowledge, Samd7/11 is the only reported a SAM domain protein with cell-type dominant expression. Even in the retina, there are not any known cell-type-specific SAM domain proteins in non-rod cells. In darkness, rod photoreceptors become sensitive to light and can ultimately respond even to a single photon^11,12^. The current ERG analysis suggests that *Samd7/11* DKO mice have reduced light sensitivity and delayed signal transmission from rods to rod bipolar cells. We think that the level of reduction in the outer segment length observed in the *Samd7/11* DKO retina might not affect the ERG amplitudes significantly. As an alternative explanation, the reduced expression of the genes involved with the phototransduction genes, including *Rhodopsin* and *Gnat1*, might affect the reduction of the ERG amplitudes observed both in the *Samd7*^*−/−*^ and *Samd7/11* DKO retinas. The mouse mutants for *Pikachurin* and *Dystroglycan*, which are synapse-related genes predominantly expressed in photoreceptors, show b-wave perturbation of reduced amplitude and an extended implicit time^13,14^. We suppose that the expression of the synapse-related molecule(s) may be affected in the *Samd7/11* DKO retina. However, we did not identify any possible synapse genes among the 136 down-regulated genes or 114 up-regulated genes in the *Samd7/11* DKO retina. We propose that rods need Samd7/11 for preventing ectopic gene expression to achieve precise gene expression, allowing for the development and maintenance of high light responsiveness in rod photoreceptors.

Taken together, the present study suggests that Samd11 interacts with Phc2 and Samd7 to be a component of PRC1 architecture and is required for correct establishment of rod photoreceptor identity. Further, it indicates that cell type-specific epigenetic factors may play an important role in establishing precise neuronal identity.

## Experimental procedures

### Animal care

All procedures conformed to the Association for Research in Vision and Ophthalmology Statement for the Use of Animals in Ophthalmic and Vision Research and were performed in compliance with institutional guidelines. The procedures were approved by the Institutional Safety Committee on Recombinant DNA Experiments (approval ID: 04220-4) and the Animal Research Committee of the Institute for Protein Research (approval ID: 29-01-3). Mice were housed in a temperature-controlled room at 22 °C under a 12 h light/dark cycle. Fresh water and rodent diet were available at all times.

### Generation of *Samd11*^*−/−*^ and *Samd7/11* DKO mice

The CRISPR/Cas9 system was used for high efficiency gene conversion. The gRNAs were designed to target the introns between exons 4 and 5 and exons 9 and 10, then cloned into the pX330 vector^15^. A MEGA short script TM T7 Transcription Kit (Ambion) and mMESSAGE mMACHINE T7 Ultra Kit (Ambion) were used to synthesize and purify the *Cas9* mRNA and *Samd11* gRNA. We cloned a 1.8 kb XhoI-ClaI fragment and a 1.6 kb EcoRI-BamHI fragment from *Samd11* genomic loci into a modified pPNT vector construct. The targeting construct was linearized and transfected into the TC1 embryonic stem cell line with *Cas9* mRNA and *Samd11* gRNAs. *Samd11*^*−/−*^ mice were crossed with *Samd7*^*−/−*^ mice^7^ to generate *Samd7/11* DKO mice.

### Electroretinogram (ERG)

2 M *Samd7*^*−/−*^, *Samd11*^*−/−*^, *Samd7/11* DKO, and WT control mice (n = 3) were used for the ERG study. The ERG recording method followed previously reported methods^16^. In brief, ERGs were recorded by a white LED luminescent electrode placed on the cornea (PuREC; Mayo, Japan). Mice were dark adapted for > 4 h, then anesthetized with an intraperitoneal injection of 100 mg/kg ketamine and 10 mg/kg xylazine. The mice were placed on a heating pad and stimulated with four stroboscopic stimuli ranging from − 4.0 to 1.0 log cd-s/m^2^ to elicit scotopic ERGs. After mice were light adapted for 10 min, they were stimulated with four stimuli ranging from − 0.5 to 1.0 log cd-s/m^2^ for photopic ERGs. The photopic ERGs were recorded on a rod-suppressing white background of 1.3 log cd-s/m^2^.

### Immunofluorescent analysis of retinal sections

Mouse eye cups were prepared using fine forceps and ophthalmological scissors, then fixed in 4% paraformaldehyde (PFA) in phosphate-buffered saline (PBS) for 5 or 30 min. The samples were then rinsed in PBS, cryoprotected in 30% sucrose/PBS overnight, embedded in TissueTek OCT compound 4583 (Sakura), frozen on dry ice, and sectioned using a MICROM HM560 cryostat (Thermo Fisher Scientific). Sections were placed on slides and dried overnight at room temperature, rehydrated in PBS for 5 min, incubated with blocking buffer (5% normal donkey serum and 0.1% Triton X-100 in PBS) for 1 h, then incubated in primary antibodies in blocking buffer overnight at 4 °C. After washing with PBS, the slides were incubated with secondary antibodies in blocking buffer for 2 h at room temperature.

The following primary antibodies were used for immunostaining: mouse anti-Pax6 (1:500, Developmental Studies Hybridoma Bank), anti-S100β (1:2,500, Sigma), anti-Ctbp2 (1:500, BD Biosciences), rabbit anti-Samd7 (1:10,000)^7^, anti-Rhodopsin (1:2,500, LSL), anti-M-opsin (1:300, Millipore), anti-Calbindin (1:1,000, Calbiochem), anti-Pikachurin (1:500)^14^, goat anti-S-opsin (1:500, Santa Cruz), anti-H3K4me3 (1:500, Millipore), guinea pig anti-Thrβ2 (1:50)^9^, anti-Samd11 (1:20,000, generated for this study), and anti-Chx10 (1:500)^17^, goat anti-Lamin B (1:250, Santa Cruz). Cy3-conjugated (1:500, Jackson ImmunoResearch Laboratories) or Alexa Fluor 488-conjugated secondary antibodies (1:500, Sigma) were used. Rhodamine-labeled Peanut Agglutinin (PNA; 1:250, Vector Laboratories) was used to stain the outer and inner cone segments and the cone synaptic terminal. DAPI (1:1,000, Nakalai) was used for nuclear staining. The specimens were observed under a laser confocal microscope (LSM700, Carl Zeiss).

### TEM (transmission electron microscope) analysis

TEM analysis was performed as previously described^18^. Mouse eye cups were fixed for 30 min with 2% glutaraldehyde and 2% PFA. The retinas were fixed with 1% osmium tetroxide for 90 min, dehydrated through a graded series of ethanol (50%–100%), cleared with propylene oxide, then embedded in epoxy resin. Sections were cut on an ultramicrotome (Ultracut E; Reichert-Jung), stained with uranyl acetate and Sato's method lead staining solution^19^, and observed under a transmission electron microscope (H-7500; Hitachi Co). Rod outer segment lengths were measured using ImageJ.

### Immunoprecipitation assay

The immunoprecipitation assay was performed as previously described^7^. The calcium phosphate method was used to transfect HEK293 cells with plasmids for 48 h. The cells were then suspended in IP250 buffer (20 mM HEPES, 10% glycerol, 250 mM NaCl, 0.2 mM EDTA, and 1 mM DTT) containing protease inhibitors (1 mM PMSF, 1 µg/ml leupeptin, 5 µg/ml aprotinin, and 3 µg/ml pepstatin A). The suspended cells were sonicated for 20 min using a Bioruptor USD-250 (Cosmo Bio). The cell lysate was then centrifuged for 10 min at 15,000 rpm. The supernatants were incubated with anti-FLAG M2 affinity gel (Invitrogen) overnight at 4 ºC. The beads were washed with IP250 buffer and eluted with FLAG-peptide (Sigma). The immunoprecipitation samples were incubated with SDS-sample buffer for 5 min at 100 ºC and analyzed by western blot analysis.

### Western blot analysis

Western blot analysis was performed as previously described^20^. Samples containing SDS-sample buffer were separated by SDS–polyacrylamide gel electrophoresis and transferred to polyvinilidine difluoride membranes (ATTO) with a semi-dry transfer cell (Bio-Rad). Chemi-Lumi One L (Nakalai, Japan) or Pierce Western Blotting Substrate Plus (Thermo Fisher Scientific) were used to detect signals. We used the following primary antibodies: mouse anti-FLAG (1:6,000, Sigma), anti-β-actin (1:5000, Sigma), rabbit anti-Samd7 (1:20,000), rat anti-HA (1:5,000, Santa Cruz), and guinea pig anti-Samd11 (1:40,000). The following secondary antibodies were used: horseradish peroxidase-conjugated anti-mouse IgG (1:10,000, Jackson Laboratory), donkey anti-rabbit IgG (1:10,000, Jackson Laboratory), anti-rat IgG (1:10,000, Jackson Laboratory), and anti-guinea pig IgG (1:10,000, Jackson Laboratory).

### Microarray analysis

Microarray analysis was performed as previously described^21^. WT control, *Samd11*^*−/−*^*,* and *Samd7/11* DKO mouse retinas were dissected at P12 from four animals in each group. Total RNA was purified with QIAzol reagent (Qiagen), amplified, and labeled with Cy3 using a Low Input Quick Amp Labeling Kit (Agilent Technologies). Hybridized arrays were scanned with an Agilent DNA Microarray Scanner (G2565A). Microarray analysis was performed by Takara Bio. The data analysis was performed with Feature Extraction software (Agilent Technologies).

### Quantitative real-time PCR (qRT-PCR) analysis

Mouse retinal total RNA was isolated for qRT-PCR using QIAzol reagent (Qiagen). The cDNA was prepared using Super Script II Reverse Transcriptase (Thermo Fisher Scientific). qRT-PCR was carried out using SYBR GreenER qPCR Super Mix (Thermo Fisher Scientific) and Thermal Cycler Dice Real Time System Single MR Q TP870 (Takara) according to the manufacturers’ instructions. The results were quantified using Thermal Cycler Dice Real Time System software version 2.0 (Takara). Gene expression levels were normalized to GAPDH expression, a housekeeping gene. The mean value for each control was set as 1.0. The primer sequences are listed in Table 1.

**Table 1:** Primer sequences.

Experiment	Primer name	sequence (5′ → 3′)
qRT-PCR	Cacna1h-F	TCTCTGAGCCTCTCACGGATACTCTGC
	Cacna1h-R	TCAGCAGGCTCCGTGTAGTCTGGGATG
	Cnga3-F	CTGGTCTTGGACTACTCTGCAGATGTC
	Cnga3-R	GAGAGACAGGATGTCCAGCTTGAAGTG
	Opn1sw-F	GCTGGACTTACGGCTTGTCACC
	Opn1sw-R	TGTGGCGTTGTGTTTGCTGC
	Gngt2-F	AACACCATTGTGTCTGGGTCCTGGA
	Gngt2-R	CAGCTCCTTCTCACTGAGGTCCTG
	Ece1-F	GAACGAAACTAGGATTGAGGAGC
	Ece1-R	GTAGTGAGCTGTGACCACCTG
	Gnb3-F	GACTTCAACTGCAATGTCTGGGAC
	Gnb3-R	ACTCTGTTGTCATGGCCAGAGAG
	Miox-F	GTGTCTTTACCACATACAAGCTC
	Miox-R	CAGCCTCCATGATGGTCATCTTC
	Nfkb1-F	TTGCCTATCTACAAGCAGAAGG
	Nfkb1-R	ACCACGCTCAGGTCCATCTC
	Rhodopsin-F	GCTTCCCTACGCCAGTGTG
	Rhodopsin-R	CAGTGGATTCTTGCCGCAG
	Gnat1-F	GCGCTTACGACATGGTGCTGGTGGAG
	Gnat1-R	CGTCGCATGTTAAGCTCCAGGAACTGC
	Cngb1-F	GAAAGTTGAACCTGAGGCTGCTG
	Cngb1-R	GTCCAGGATTAGGCTGGGAG
	Gnb1-F	TCTGGTGCTTGTGATGCTTCAG
	Gnb1-R	AGGTCAAACAGCCTGCATGTG
	Gucy2f.-F	GAATTCGAATAGGCCTGCACTCG
	Gucy2f.-R	CCCTGTTGATTCCATTCGAGAAGC
	Esrrb-F	TACCTGAACCTGCCGATTTCCCCA
	Esrrb-R	AGGCATAGCATACAGCTTGTCCTG
RT-PCR	b-actin-F	CGTGCGTGACATCAAAGAGAA
	b-actin-R	TGGATGCCACAGGATTCCAT
	Samd7-F	CGACTCAGGAGAGGTGCAGTTTACCAG
	Samd7-R	TCTGAGCAGCCTGGAAGGCTTCTGATG
	Samd11-F	TCCCAGTGGCTTGGAAGTTCACCT
	Samd11-R	GAGATGGGAGCTCATTGTGATGTG

### Antibody production

A cDNA fragment encoding a middle portion of mouse Samd11 (residues 52–266) was amplified and subcloned into pET28 plasmid (Novagen). The His-tagged Samd11 fusion protein was expressed in E. coli (BL21-DE3) and purified using Ni–NTA Agarose (QIAGEN). The beads that absorbed His-tagged proteins were washed with wash buffer 1 [50 mM NaH_2_PO_4_, 300 mM NaCl, and 10 mM imidazole (pH 8.0)] and wash buffer 2 [50 mM NaH_2_PO_4_, 300 mM NaCl, and 20 mM imidazole (pH 8.0)]. Then proteins were eluted with elution buffer [50 mM NaH_2_PO_4_, 300 mM NaCl, and 250 mM imidazole (pH 8.0)]. The anti-Samd11 antibody was raised by immunizing guinea pig with the purified fusion protein.

### Statistical analysis

Data are presented as means ± standard deviation (SD). Statistical significance was assessed using Student’s t-tests. **P < 0.01 or *P < 0.05 was considered statistically significant.

## Supplementary Information

Supplementary Figures.
