# Supplementary material for: Functional analysis of Samd11, a retinal photoreceptor PRC1 component, in establishing rod photoreceptor identity
Source: Sci Rep. 2021 Feb 18;11:4180. doi: 10.1038/s41598-021-83781-1 (PMC7892874; doi:10.1038/s41598-021-83781-1)
Supplement: Supplementary file 1 — Supplementary Figures. [file 41598_2021_83781_MOESM1_ESM.pdf]

Supplementary Information

Functional analysis of Samd11, a retinal photoreceptor PRC1 component, in establishing rod photoreceptor identity

Shun Kubo[1], Haruka Yamamoto[1], Naoko Kajimura[2], Yoshihiro Omori[1], Yamato Maeda[1], Taro Chaya[1], Takahisa Furukawa[1*]

[1]Laboratory for Molecular and Developmental Biology, Institute for Protein Research, Osaka University, Osaka, 565-0871, Japan
[2]Research Center for Ultrahigh Voltage Electron Microscopy, Osaka University, Osaka, 567-0047, Japan

*Corresponding author: Takahisa Furukawa
E-mail: takahisa.furukawa@protein.osaka-u.ac.jp
Laboratory for Molecular and Developmental Biology, Institute for Protein Research, Osaka
University, 3-2 Yamadaoka, Suita, Osaka, 565-0871, Japan
Phone: +81-6-6879-8631
Fax: +81-6-6879-8633

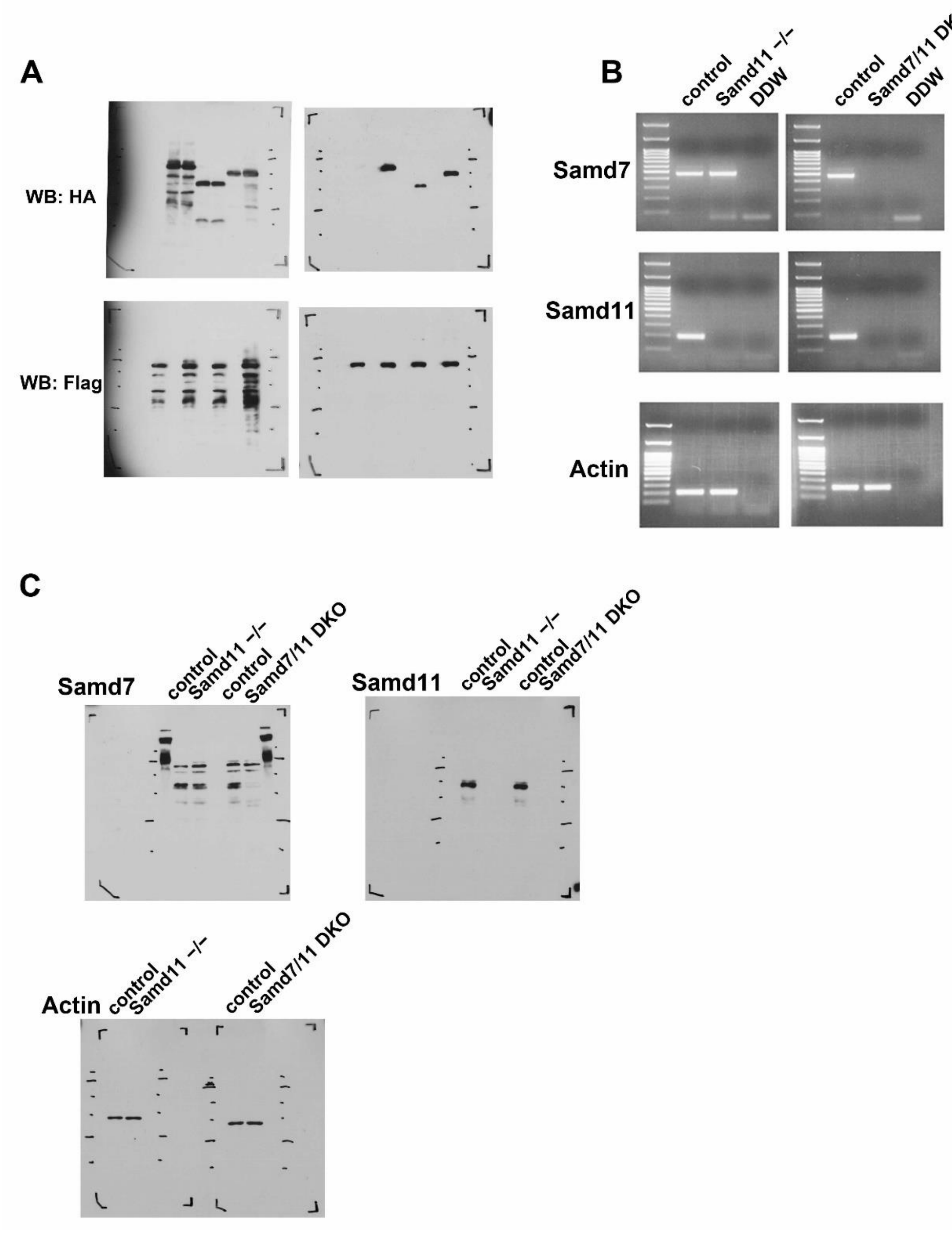

Figure S1. Full images of immunoprecipitation, RT-PCR, and Western blot analyses.

A, Full images of the immunoprecipitation analysis of Samd11/Samd11, Samd11/Phc2, and Samd11/Samd7.

B, Full images of the RT-PCR analysis of Samd7 and Samd11 transcription using intron-spanning primer sets in *Samd11*$^{-/-}$ and *Samd7/11* DKO retinas at P12.

C, Full images of the Western blot analysis of Samd7, Samd11, and Actin proteins in control, *Samd11*$^{-/-}$, and *Samd7/11* DKO retinas at P12.

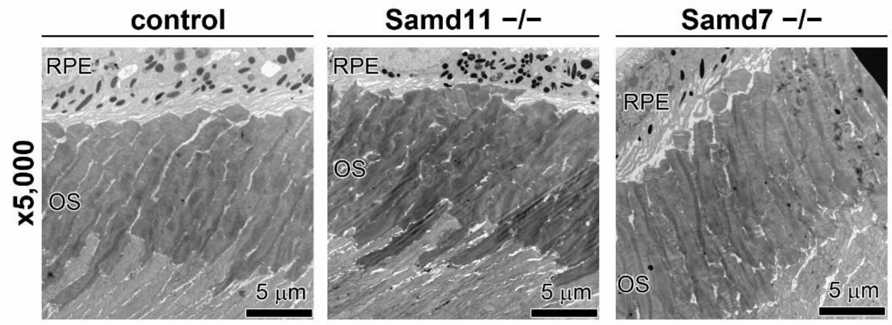

Figure S2. TEM analysis of photoreceptor outer segments.
TEM images of the outer segments in the control, *Samd11*$^{-/-}$, and *Samd7*$^{-/-}$ retina at the adult stage.

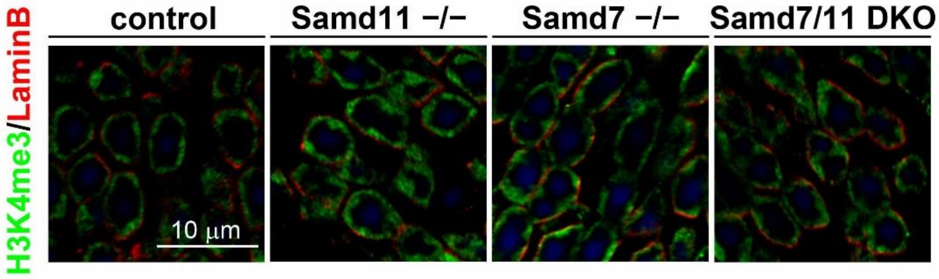

Figure S3. Immunostaining of H3K4me3 and Lamin B in the control, *Samd11*$^{-/-}$, *Samd7*$^{-/-}$, and *Samd7/11* DKO photoreceptor cells at the adult stage.

Retinal sections from control, *Samd11*$^{-/-}$, *Samd7*$^{-/-}$, and *Samd7/11* DKO mice were immunostained with anti-H3K4me3 (a marker for euchromatin) and anti-Lamin B (a marker for the nuclear membrane) antibodies.
